# Leveraging Neural Networks in Preclinical Alcohol Research

**DOI:** 10.3390/brainsci10090578

**Published:** 2020-08-21

**Authors:** Lauren C. Smith, Adam Kimbrough

**Affiliations:** 1 Department of Psychiatry, School of Medicine, University of California San Diego, MC 0667, La Jolla, CA 92093, USA; las005@ucsd.edu; 2 Department of Basic Medical Sciences, College of Veterinary Medicine, Purdue University, 625 Harrison Street, West Lafayette, IN 47907, USA

**Keywords:** addiction, dependence, substance use disorder, iDISCO, fMRI, modularity, graph theory, animal model, binge drinking, alcohol use disorder

## Abstract

Alcohol use disorder is a pervasive healthcare issue with significant socioeconomic consequences. There is a plethora of neural imaging techniques available at the clinical and preclinical level, including magnetic resonance imaging and three-dimensional (3D) tissue imaging techniques. Network-based approaches can be applied to imaging data to create neural networks that model the functional and structural connectivity of the brain. These networks can be used to changes to brain-wide neural signaling caused by brain states associated with alcohol use. Neural networks can be further used to identify key brain regions or neural “hubs” involved in alcohol drinking. Here, we briefly review the current imaging and neurocircuit manipulation methods. Then, we discuss clinical and preclinical studies using network-based approaches related to substance use disorders and alcohol drinking. Finally, we discuss how preclinical 3D imaging in combination with network approaches can be applied alone and in combination with other approaches to better understand alcohol drinking.

## 1. Introduction

Alcohol abuse is a pervasive societal problem with substantial socioeconomic and medical consequences [1]. Alcohol use disorder (AUD) is a chronic relapsing disorder that is associated with the loss of control of ethanol drinking and compulsive drinking that is driven by the emergence of a negative affective state upon cessation [2,3]. The transition from casual drinking to alcohol dependence is thought to occur over time as the motivation to drink shifts from positive reinforcement to avoiding negative reinforcement [4]. Once dependent, three repeating stages are postulated to occur. The first stage being a period of excessive drinking, followed by a second stage involving a period of abstinence, resulting in negative affect symptoms, which then leads to the third stage that involves increased motivation and craving to drink [3,5,6].

In humans, the emotional and physical signs of withdrawal from alcohol can manifest in several ways such as increased anxiety, increased irritability, increased frustration, increased aggression, mood swings, insomnia, tremors, convulsions, higher blood pressure, accelerated pulse, accelerated breathing, dehydration, and delirium tremens [7,8,9,10,11,12,13,14]. The emotional symptoms of withdrawal from alcohol dependence, including anxiety-like behavior, depression-like behavior, irritability-like behavior, and aggressive behavior can be modeled in rats and mice and can last for days to weeks after cessation of drinking [15,16,17,18,19,20,21,22,23,24,25,26,27,28,29,30,31,32,33,34,35,36,37,38,39].

Preclinical animal models, especially in rats and mice, are ideal for studying alcohol drinking and alcohol addiction due to the ability to adequately model negative affect and excessive drinking behavior. Some of the most prominent animal models to study excessive and binge-like drinking are the intermittent access to ethanol (IAE) model, using two-bottle choice [40,41,42,43,44,45,46,47,48,49,50,51,52,53,54,55,56,57,58,59,60], and the drinking in the dark (DID) model, using a single bottle [61,62,63,64,65,66,67,68,69,70,71,72]. For a comprehensive review of the behavioral methods used to study AUD in rodents, see Vendruscolo and Roberts and Tunstall et al. [73,74].

Rats and mice have also been selectively bred to express excessive drinking behavior, allowing for greater focus on genetic aspects associated with increased drinking. In rats, the alcohol-preferring strain, the high alcohol-drinking strain, and the Marchigian Sardinian alcohol-preferring rats have been examined [75,76,77,78,79], and in mice, high drinking in the dark mice and high alcohol-preferring mice have been examined [80,81,82,83,84,85].

To study excessive and compulsive-like drinking due to alcohol dependence, the intermittent ethanol (CIE) vapor exposure model has been heavily used in both rats and mice [16,55,86,87,88,89,90,91,92,93,94,95,96,97,98,99,100,101,102,103,104,105].

Although our knowledge of the neurocircuitry that underlies excessive alcohol drinking and alcohol dependence has improved dramatically over the last decade [5,106], there is still a large need to further our understanding. Adequately advancing our understanding of the neurocircuitry involved in alcohol drinking will require research to continue to take advantage of quality preclinical models and emerging technological advancements in the field of neuroscience.

## 2. Recently Developed Approaches in Preclinical Neuroscience

The development of site-specific recombinase systems [107,108,109] has allowed for controlled mutations in preclinical models of disease [110]. Advances in cell-specific molecular genetics has built the foundation for the modern optogenetic, chemogenetic, and electrophysiology methods, used in cell-specific populations. 

### 2.1. Optogenetics

The optogenetic method takes advantage of the genetic incorporation of light-gated cation channel channelrhdopsin-2 (ChR2) and chloride and proton pumps (i.e., halorhodpson and archaerhopsin) into neural tissue [111,112,113]. Neuron specific Cre recombinase expression allows for the use of viral vectors encoding Cre-inducible opsin proteins in specific brain regions [114]. Optogenetics can be used to map neural connections between specific sets of brain regions. Optogenetic manipulation can further reveal distinct circuits involved in behavioral and emotional states such as reward, stress, and anxiety-like behaviors [115].

Optogenetic approaches in preclinical models have been used to identify numerous circuits throughout the brain involved in alcohol drinking. Several studies have used optogenetic manipulation to identify projections from the central amygdala (CEA) that are critical for addiction-like behaviors [116,117,118]. Optogenetic manipulation of ventral tegmental area (VTA) neurons projecting to the nucleus accumbens (NAc) has identified a role for the VTA–NAc circuit in alcohol-drinking behavior [119,120,121]. Other circuits determined to be important for alcohol-related behaviors via optogenetics include an agranular insular cortex to basolateral amygdala (BLA) circuit [122] and a medial prefrontal cortex (mPFC) to NAc circuit. Interestingly, inhibition of the mPFC–NAc circuit was found to reduce alcohol drinking associated with quinine adulteration, suggesting the importance of the circuit for compulsive-like alcohol drinking [123]. While optogenetic approaches allow for precision in targeting neural circuits, they require surgical manipulation and active stimulation to produce inhibition or activation. Therefore, the approach examines the inhibition or activation of circuits for a period of seconds to minutes but not days to weeks, which may be needed to determine long-term effects. Optogenetics has been used to make great progress toward understanding the circuits mediating alcohol-related behavior and will likely continue to be an important approach in neuroscience. Chemogenetics are a complementary set of approaches that have similarly been used to explore circuits related to alcohol drinking.

### 2.2. Chemogenetics

Designer receptors exclusively activated by a designer drug (DREADDs) are a class of chemogenetically engineered receptors activated by small molecules [124]. DREADDs are engineered G-protein coupled receptors that have been modified to be activated exclusively by synthetic compounds (e.g., clozapine-N-oxide). The use of DREADDs serves as a less invasive and more affordable alternative to optogenetic approaches. The expression of DREADDs in specific brain regions is usually achieved with stereotaxic injection of an Adeno-associated virus encoding Cre or flippase DNA recombinases. The development of transgenic DREADD reporter mice for Cre- or Flp-driven chemogenetic manipulation allows for brain-wide, cell-specific circuit investigation [124,125,126]. Several DREADD variants have been engineered using a G-protein coupled receptor or β-arrestin signaling components [125,127,128]. Similar to optogenetics, chemogenetics has been used to identify distinct neural circuits associated with behavioral and emotional states [127,129]. 

Several studies have taken advantage of chemogenetics to explore mechanisms of alcohol drinking. For example, a reduction of alcohol consumption in mice was found by a suppression of activity in the NAc core with hM4Di-mediated inhibition [130]. Pharmacological antagonism of the k-opioid receptor and chemogenetic silencing of dynorphin signaling neurons was used to identify a role for dynorphin/k-opioid receptor signaling in the central amygdala in excessive alcohol consumption in a rodent model of binge drinking [131]. Specific neuronal ensembles critical for alcohol dependent drinking within the CEA were identified by the chemogenetic silencing of activated neurons using Daun02 [91]. Inhibition of dorsal mPFC projections to the BLA via chemogenetics reduced withdrawal symptoms associated with abstinence for alcohol dependence [132]. With chemogenetic methods, receptors are targeted via the injection of a ligand, resulting in a less specific time of effect. Furthermore, some ligands may produce potential off-target effects depending on the dose used, thus requiring ligand-only controls [133,134,135,136]. Although optogenetics and chemogenetics tend to focus more on neural circuits, other techniques such as calcium imaging are necessary to determine the activity of individual neurons over time in a single brain region. 

### 2.3. Calcium Imaging

Two-photon calcium imaging is a method for monitoring the in vivo activity of distinct neurons in brain tissue [137]. This allows for a real-time analysis of cells and subcellular compartments. Calcium imaging can also be used in in vitro studies in brain slices [138]. The combination of acetoxymethyl ester staining and calcium imaging techniques allows for the characterization of neural network activity on a larger scale [139]. Calcium imaging has been used to identify neural circuits involved in behaviors mediating substance use disorders (SUD) and AUD. For example, calcium imaging was able to identify neural activity patterns in mPFC neurons projecting to the periaqueductal gray that predicted the emergence of compulsive-like alcohol drinking behavior [140]. Calcium imaging is able to assess activity patterns of individual neurons; however, this approach is often limited to brain regions closer to the surface of the skull and does not examine the neural activity of the brain as a whole. Calcium imaging can also be performed using fiber photometry, which allows for access to deeper brain regions. 

### 2.4. Magnetic Resonance Imaging

One of the most commonly used neuroimaging techniques is magnetic resonance imaging (MRI), which is relatively fast, non-invasive, and can be done in vivo. MRI takes advantage of the ability of atomic nuclei to absorb radio frequency (RF) in the presence of an external magnetic field [141]. Then, the nuclei emit an RF signal with intrinsic spin polarization that can be detected in a radio frequency coil. This method has evolved over time, and advanced applications are currently available to look at functional brain activity in human subjects and preclinical animal models [142,143]. Functional magnetic resonance imaging (fMRI) is a method that measures time-based changes in brain metabolism, which are functionally measured as changes in oxygenated blood during a neural response [143]. While MRI techniques are not a recent development, their application in determining structural and functional connectivity is a relatively new advancement in the field of neuroscience. Magnetic resonance imaging methods are useful to measure within-subjects; however, they require a head-fixed and anaesthetized state. Furthermore, imaging is only possible at a mesoscale resolution. Neural activity can be examined in preclinical animal models by using fMRI; however, there are limitations on the resolution of the activity patterns that may limit the usefulness in rodent models of disease.

### 2.5. Three-Dimensional Imaging

Recent developments in tissue-clearing methods allow for three-dimensional (3D) imaging of intact tissues, such as the whole brain. Hydrophobic and hydrophilic tissue-clearing methods are both solvent-based and generally remove lipids, pigments, and calcium phosphate to reach an appropriate refractive index for imaging. The 3D imaging of tetrahydrofuran (THF) cleared organs (3DISCO) was developed by Ertürk et al. The use of THF, a dehydrating and de-lipidating agent, instead of alcohol enhances the refractive index homogeneity of the samples [144]. Renier et al., developed the immunolabeling-enabled DISCO (iDISCO) method, which allows for whole-mount immunolabeling of whole cleared organs [145]. This technique was adapted and improved by removing the use of THF and instead only using methanol to dehydrate the tissue with iDISCO+ [146]. The ‘ultimate’ DISCO (uDISCO) technique, developed by Pan et al., was optimized for the preservation of endogenous fluorescence for months [147]. Hydrogel-based tissue clearing uses covalent linkage to an acryl-based hydrogel for complete lipid removal with limited structural damage and protein loss [148]. This method has been termed ‘cleared lipid-extracted acryl-hybridized rigid immunostaining/in situ hybridization-compatible tissue hydrogel’, or CLARITY. A summary of 3D imaging techniques and some of the pros and cons of each method are presented in Table 1. The availability of many 3D imaging methods allows researchers to tailor the method for a given experiment, each with their own advantage or disadvantage. With all 3D imaging methods, only between-subject timepoints are possible, as the method requires post-mortem data collection. Neural activity measured by 3D imaging can be immense and hard to interpret without combining the technique with network neuroscience to identify brain-wide neural activity at the network level. 

## 3. Neural Networks

Network-based approaches are highly applicable to neuroscience and have been used to analyze large sets of data. In neuroscience, networks can be examined at the structural or functional connectivity level. Structural networks are mapped by measuring the physical inter-neuronal connectivity or inter-regional connectivity, whereas functional networks examine the effective connectivity of neural activity between regions [151]. Methods for looking at structural connectivity include MRI, diffusion tensor imaging, and electron microscopy [152,153,154,155]. Functional connectivity can be measured using fMRI, electroencephalography (EEG), magnetoencephalography (MEG), or 3D imaging [156,157,158,159,160,161,162,163,164,165,166]. A summary of the methods used to model the functional and structural connectivity of the human brain is presented in Table 2.

Graph theory has been used to identify specific features of neural networks in more detail. Graph theory is a branch of mathematics that is used to analyze complex networks [129,168,169,170,171,172,173,174,175,176,177,178,179]. Graph theory can be applied to data across multiple levels of time and neural organization (e.g., whole brain, regions, circuits, neurons). Graph theory models the pairwise relations between vertices, or nodes, through interconnecting edges, which can be directed or undirected [180]. When modeling the brain, the node can be neurons or an anatomical brain region, and the edges can be the functional connectivity between them, as measured by the correlation of neural activity.

In terms of brain mapping, numerous studies have examined the human brain (Table 2). Many of the graph models of human brain imaging data show small-worldness and modular organization [164,181]. In graph theory, the “world” of a network is said to be “small” if the mean geodesic distance between node pairs is small relative to the total number of nodes. The geodesic distance is calculated by determining the minimum number of edges required to travel between two nodes [182]. Mathematically, as the number of nodes tends to infinity Equation (1), the mean geodesic (g) will grow slower than logarithmically Equation (2).

(1) N→∞

(2) g=r(logN)

Modularity is defined as the division of a network into modules. A network expressing high modularity consists of nodes with high intramodule connectivity and sparse intermodule connectivity [181]. Mathematically, modularity is calculated by taking the fraction of edges that fall within modules minus the expected fraction of edges. These topographical measures translate across methods and correlate with phenotypes and disease states. For example, in patients with schizophrenia, network analysis of EEG and fMRI data shows changes in the magnitude of connectivity across brain regions relative to healthy controls [183,184]. Additionally, general intelligence has been shown to be associated with topological measures of network efficiency in neural network analysis of functional and structural brain mapping data [185,186,187]. Graph theory can further be used to determine network features such as network efficiency and node centrality. Network efficiency is characterized by the average of the shortest path lengths between any pair of nodes, with lower values indicating higher efficiency [164,188]. Node centrality quantifies the importance of a node inside a network and can consider degree, efficiency [158], closeness, or betweenness [189]. These nodes are considered a hub in the network [190,191].

In modeling neural networks, hub brain regions will emerge that are characteristic of the specific brain state being examined. A hub is defined as a node with high connectivity to other nodes in the same module (provincial hubs) or to other modules (connector hubs) [191,192]. Hub brain regions are critical to network function and represent the highest level of connectivity [99,129,171,190,193]. An example of hub identification and validation in brain-wide neural networks can be seen in a study by Vetere et al., in which in silico node deletion and in vivo chemogenetic silencing of the identified nodes confirmed the connection between a region’s node degree and role in memory consolidation [129]. Furthermore, important hubs identified in neural networks have been shown to be conserved across species and scales [194]. Going forward, network neuroscience has the potential to provide novel insights regarding how the brain functions as a whole in brain states associated with SUDs.

## 4. Neural Networks in Clinical Models of Substance Use Disorders

There have been attempts to identify modules and neurocircuitry involved in SUDs from large-scale analysis of prior literature [106,195]. However, the majority of the SUD studies leveraging neural network analysis have involved imaging data. Differential levels of functional connectivity in alcohol, tobacco, and concurrent alcohol and tobacco users compared to a control population that did not use substances have been determined by resting-state fMRI analysis [196]. The results showed a general reduction in functional connectivity among substance users, with hyper-connectivity to the precuneus observed in smokers. Smokers examined during abstinence, and after satiation, using whole brain resting-state fMRI were found to have functional neural adaptations in the anterior cingulate cortex and precuneus during withdrawal-induced craving [197]. The precuneus is involved in a variety of functions, including memory [198] and has been proposed as a core component of the default mode network [199]. The use of fMRI, combined with memory and cognition tasks, after administration of the psychostimulant methylphenidate in healthy individuals was used to identify key brain regions involved in regulating cognition [200,201]. Analysis of resting-state fMRI data from cocaine-dependent individuals after treatment with methylphenidate was used to identify connectivity patterns in the mesocorticolimbic system [202]. Studies of brain connectivity during cocaine dependence using MRI techniques have shown that cocaine use is associated with altered brain connectivity that is associated with behavioral performance, treatment outcomes, and history of use [203]. Cocaine use has also been found to alter the identification of brain hubs in neural networks [204]. Abstinent heroin-dependent users experienced increased impulsivity and greater intrinsic amygdala functional connectivity at resting state compared to controls [205]. Abstinence from heroin dependence has also been shown to decrease the resting-state functional connectivity of the anterior cingulate cortex, which was associated with cue-induced cravings for heroin [206]. 

Alcohol-dependent individuals and binge drinkers have been shown to have major alterations to functional connectivity in several studies. Resting-state functional connectivity in executive control brain regions has been found to be reduced in subjects suffering from AUD [207,208]. Acute alcohol intake was found to result in greater changes to functional connectivity in heavy drinkers than normal drinkers [209]. Increased functional connectivity has been observed in patients with AUD during acute withdrawal in task-based cue-reactivity fMRI studies, identifying brain regions involved in craving [210,211]. This was accompanied by cue-based functional dysconnectivity and resting-state hyperactivity in specific cortical and subcortical regions as measured by EEG [210]. This study supports the hypothesis of a network of alcohol-related brain areas connected to craving, including the amygdala, parahippocampus, NAc, striatum, posterior cingulate cortex, and VTA. Similarly, young adult binge drinkers were found to have greater connectivity between striatal areas associated with reward, such as the NAc, and salience-associated areas, such as the anterior cingulate cortex, and reduced connectivity of the prefrontal cortex and hippocampus [212]. Alcohol has been shown to reduce connectivity between the globus pallidus externus (GPe) and other brain regions in patients with history of alcohol use. In the same study, impulsivity was correlated with greater GPe connectivity during intoxication, and the contribution of drinking and impulsivity to GPe connectivity were found to be distinct [213]. Taken together, these highlighted studies indicate that major changes occur in the brain after using substances of abuse such as alcohol. However, there are limitations to the amount of detail that can be inferred from human studies; thus, there is a need to broaden neural network approaches to animal models of SUD.

## 5. Leveraging Neural Networks in Preclinical Animal Models

**Figure 1:**
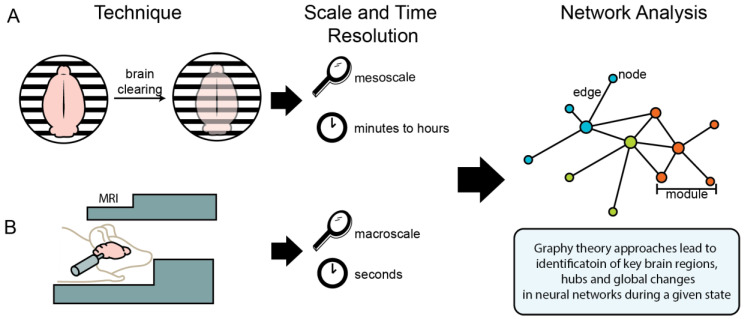
Illustration of imaging techniques used for neural network analysis of functional and structural brain connectivity. (**A**) Single-cell whole-brain imaging techniques, such as iDISCO, allow for analysis of the whole brain at the mesoscale (i.e., with region and cell-specific resolution), with results representing neural activity across minutes to hours. (**B**) Magnetic resonance imaging (MRI) techniques allow for analysis of the brain in more generalized resolution (i.e., at the macroscale) in anesthetized or immobilized animals, with results representing neural activity across seconds. Data from both of these methods can be interpreted using graph theory approaches to identify key brain regions, hubs, and global changes in neural networks during a given state.

Network-based approaches can also be applied to preclinical animal models using imaging techniques such as fMRI, traditional immunohistochemistry, and whole-brain single-cell imaging (i.e., iDISCO). Figure 1 summarizes two of the available preclinical imaging techniques and their application to neural networks. Whole-brain single-cell imaging can capture a cognitive state (e.g., alcohol abstinence) at a single point in time, at a mesoscale resolution. *Fos* protein begins to be produced at detectable levels approximately 30 min after neural activity, and peaks at 60 to 90 min, and returns to baseline at approximately 180 min; thus, the data from *Fos* protein represents neural activity across minutes to hours. This method allows immunostaining for multiple proteins and combination with other approaches such as optogenetics, chemogenetics, or pharmacology. This approach allows for data to represent normal awake-behaving neural activity, allowing for multiple experimental paradigms to be explored. Some limitations to single-cell whole-brain imaging are that it is ex vivo and only samples a single timepoint. In contrast, fMRI can capture a time course within the same subjects in vivo with data representing neural activity across seconds per measurement. Some limitations to fMRI are lack of brain region specificity in the data and that the animals are either immobilized or anesthetized during sampling, which prevents assessing many experimental paradigms (Table 3).

### 5.1. Preclinical Studies of Substance Use Disorders Using Functional Magnetic Resonance Imaging

Functional MRI has been used in preclinical models of neurological disease and SUD to identify general trends in functional connectivity, as well as key brain regions and neural pathways involved in these states. Compared to controls, the thalamus was found to be heavily involved in the neural network activity of rats in acute abstinence from cocaine self-administration. Interestingly, changes in the neural network seen by fMRI measurement disappeared after 2 weeks [214]. A longitudinal study in a rodent model of alcohol use disorder utilized resting-state fMRI during a baseline measurement and after chronic alcohol use to determine a brain network and functional connectivity alterations associated with excessive alcohol drinking. An overall decrease in brain functional connectivity after chronic alcohol use and increased functional connectivity between the striatal and prefrontal–cingulate were found [215]. Although preclinical fMRI data can help to better understand brain function associated with SUD, the lack of resolution with regard to brain region specificity suggests that other approaches may be more beneficial in preclinical network neuroscience.

### 5.2. Preclinical Studies of Substance Use Disorders Using Single-Cell Whole-Brain Imaging

An unbiased approach to examine whole-brain neural activity by light-sheet fluorescent imaging of immunostained and optically cleared tissue has recently been developed (iDISCO) and used to uncover brain regions differentially activated during parenting behavior [145,146]. Another approach identified brain-wide maps of *Fos* mRNA expression during auditory fear conditioning to reveal patterns of *Fos* induction that are similar among shock-only and tone-only fear conditioning and fear recall conditions, suggesting simple associative learning ensembles that are activated by arousal rather than by a specific sensory cue [216]. 

Recent studies have begun to use protein detection of the immediate early gene *Fos* in combination with functional connectivity and network analysis. The quantification of 81 brain regions for *Fos* protein, using traditional immunohistochemistry, after fear conditioning led to the identification of a fear network [171]. This fear network was further examined using in vivo chemogenetic silencing of different network nodes to confirm the importance of the hubs predicted in network models [129]. Similarly, an unbiased brain-wide *Fos* protein approach, using the single-cell whole-brain imaging (iDISCO+) method, has been combined with functional connectivity and graph theory to identify changes to neural network structure and function caused by withdrawal from alcohol and psychostimulants [99,217].

Neural networks associated with withdrawal from individual psychostimulants (cocaine, methamphetamine, and nicotine) were identified in mice by using single-cell whole-brain imaging of neural activity [217]. While withdrawal from each drug produced a distinct pattern of brain activity, methamphetamine and cocaine had the most overlapping similarities. The common neuroadaptation between these psychostimulants was not necessarily changes in the connectivity of a specific group of brain regions, but an overall decrease in the modularity of the network. Global adaptations in the functional networks, such as decreased modularity, have been observed in other neuropsychiatric disorders, such as dementia, seizures, and traumatic brain injury [181,218,219,220,221,222].

**Figure 2:**
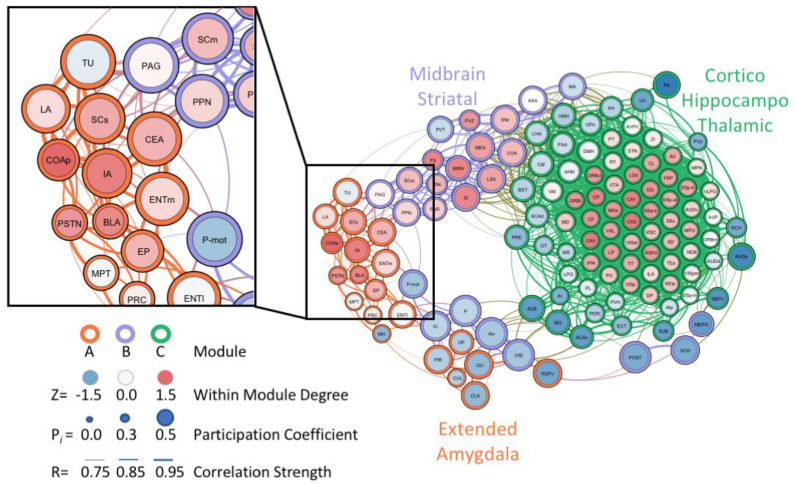
Graph theory representation of the neural network associated with alcohol abstinence in alcohol-dependent mice. Analysis identified several underexplored regions within the extended amygdala module that may be critical for withdrawal-associated behavior and excessive alcohol drinking. The zoomed-in panel highlights several of the underexplored regions, which include the posterior cortical amygdala (COAp), intercalated amygdala (IA), parasubthalamic nucleus (PSTN), and tuberal nucleus (TU). Figure reproduced and modified from Kimbrough et al., 2020 *PNAS*.

Similarly, the unbiased single-cell whole-brain imaging and network analysis approach identified a massive increase in coactivation among brain regions and reduced modular structuring of the brain during abstinence from alcohol dependence when compared to control networks [99]. In the alcohol abstinence network, an overall structural simplification was observed, with three large modules identified that corresponded well to the classic three-stage theory of addiction [3,5,6]: an extended amygdala module, a midbrain striatal module, and a cortico-hippocampo-thalamic module. This approach was able to verify the importance of regions within the extended amygdala that are known for their involvement in alcohol drinking and withdrawal, such as the CEA [23]. Additionally, brain regions of interest within the extended amygdala module, which may have been previously overlooked, were identified as targets for future research (Figure 2). These regions included the parasubthalamic nucleus, tuberal nucleus, cortical amygdala, and intercalated amygdala [99]. The findings related to alcohol abstinence [99] represent a small portion of the ways that whole-brain imaging and neural network-based approaches can be leveraged to ask and answer questions in the alcohol field going forward.

### 5.3. Ways to Use Network-Based Approaches in the Preclinical Alcohol Field Going Forward

The network-based approach using single-cell whole-brain imaging provides a unique opportunity to assess different aspects of alcohol drinking and AUD through various preclinical models (e.g., binge-like drinking, casual drinking etc.) and states of drinking/dependence depending on the question of interest. The different preclinical behavioral paradigms models (e.g., IAE, DID, CIE) of alcohol drinking and alcohol-preferring rodent strains can be used to assess neural networks across severities and conditions of alcohol drinking (e.g., binge drinking, alcohol dependent drinking, abstinence, etc.). Similarities and differences in brain-wide networks can be compared across alcohol drinking paradigms, and the unique components of a given network can be identified.

Perhaps most exciting of all is the possibility to combine single-cell whole-brain imaging and network analysis with other recently developed preclinical neuroscience technology. For example, the Targeted Recombination in Active Populations (TRAP) technique, in which mice contain a tamoxifen-dependent recombinase CreER^T2^ that is expressed in an activity-dependent manner from the loci of the immediate early genes *Arc* and *Fos.* This allows for the labeling of activated neurons at a given time/behavioral state (e.g., after a discrete drinking session, during intoxication, during withdrawal, etc.) while maintaining the ability for further testing before collection of the brain to examine neural networks [223,224]. Then, TRAP *Fos* labeling from a previous timepoint of neural activity (e.g., intoxication) can be combined with traditional *Fos* immunostaining to mark for natural immediate early gene protein production, using iDISCO, which is associated with neural activity from a time window shortly prior to euthanasia (e.g., protracted abstinence). Then, the neural activity from these two timepoints could be used together to assess neurons that are activated at both timepoints and potential similarities and differences in neural network activity between the two timepoints.

Preclinical network analysis using immediate early gene immunofluorescence can also be combined with immunostaining or endogenous fluorescence that signals specific neuronal cell types. This approach will provide information on a wide scale about what types of neurons and receptors contribute to overall network activity. Furthermore, viral tracing between brain regions can determine structural connections and then be used to compare structural to functional connectivity within a given network.

Optogenetics or DREADDs can be combined with single-cell whole-brain imaging to explore the effect of manipulation of specific circuits or cells on the neural connectivity of the whole brain. This approach would be ideal for delineating regions that play a role in a given behavior associated with the specific circuit but that is at the tertiary, quaternary, or further level of separation from the stimulation/inhibition. Additionally, using optogenetics or DREADDs to disrupt the activity of brain regions identified by network analysis combined with whole-brain imaging as “hub” regions associated with a behavioral state can verify their functional importance for the behavior. Calcium imaging can also be used to uncover more specific neuron-to-neuron local networks within a given brain region alongside an examination of brain-wide networks in the same animals. There is also the potential to use multi-brain region fiber photometry to collect neural activity data from neurons across a large number of brain regions [225], either independently, or as a complementary approach alongside single-cell whole brain imaging.

Traditional pharmacology with drug candidates that are potential medications to treat alcohol drinking can be explored as well using neural network approaches. It would be interesting to know how efficacious a given drug is at restoring the network function of the brain to a more normal state after a history of drinking. Perhaps current medications for the treatment of AUD, such as the FDA-pproved naltrexone, acamprostate, and disulfiram, only relieve changes to brain-wide network activity partially, or for a short period of time, which may explain to some degree the limited/moderate efficacy [226]. Interestingly, there is evidence that naltrexone alters functional connectivity in humans. Naltrexone was found to normalize local network efficiency in alcohol-dependent subjects [227]. Additionally, enhanced functional connectivity between the cingulate cortex and prefrontal cortex has been identified as a potential key component of the mechanism of action of naltrexone to treat alcohol drinking [228]. Overall, the preclinical field of alcohol addiction can benefit tremendously by taking advantage of single-cell whole-brain imaging and neural network approaches over the next several years.

## 6. Conclusions

Network-based approaches in preclinical studies have the potential to make significant contributions to our understanding of alcohol drinking. The combination with other relevant neuroscience approaches provides a unique opportunity to enhance our systems-based understanding of the brain in a way that was not previously available. Further, network analysis may help improve medication development and lead to better therapeutic options for AUD.

## Figures and Tables

**Table 1:** Three-dimensional (3D) imaging techniques. 3DISCO: 3D imaging of tetrahydrofuran (THF) cleared organs, iDISCO: immunolabeling-enabled DISCO, uDISCO: ‘ultimate’ DISCO, CLARITY: cleared lipid-extracted acryl-hybridized rigid immunostaining/in situ hybridization-compatible tissue hydrogel.

Imaging Technique	Clearing Method	Pros	Cons	Reference
3DISCO	tetrahydrofuran and dibenzyl ether	relatively quick tissue clearing, allows for 3D imaging of entire intact tissue	can only be used on fixed tissue, cannot store tissue for long periods of time	[149]
iDISCO	tetrahydrofuran and dibenzyl ether	allows for whole mount immunolabeling	can bleach endogenous fluorescent signals	[145]
iDISCO+	methanol and dibenzyl ether	allows for whole mount immunolabeling	can bleach endogenous fluorescent signals	[150]
uDISCO	diphenyl ether, benzyl alcohol, benzyl benzoate, and α-tocopherol	preserves endogenous fluorescent signal, reduces volume, virus labeling, and immunostaining compatible	significant shrinkage of tissue	[147]
CLARITY	acrylamide and bisacrylamide hydrogel, formaldehyde, and thermal transduction	suitable for long-stored organs, preserves endogenous fluorescence signal	not ideal for immunostaining of the brain	[148]

**Table 2:** Methods used for human brain mapping.

Method	Domain	References
functional magnetic resonance imaging	Functional	[156,157,158,159,160,161]
electroencephalography	Functional	[162,163]
magnetoencephalography	Functional	[165,166,167]
diffusion tensor imaging	Structural	[152]
diffusion spectrum imaging	Structural	[153]
magnetic resonance imaging	Structural	[154,155]

**Table 3:** Pros and cons of imaging techniques used for neural network analysis of functional and structural brain connectivity.

Technique	Pros	Cons	References
Single-cell whole-brain imaging	mesoscale resolution (i.e., with region and cell-specific), organs taken in the freely moving state	single timepoint, post-mortem measurements	[145,147,149,150]
MRI and fMRI	repeatable measurement for multiple timepoints within-subject	anesthetized or immobilized during measurement, generalized brain region resolution (i.e., at the macroscale)	[141,142,143,189]
